# The Environmental Light Characteristics of Forest Under Different Logging Regimes

**DOI:** 10.1002/ece3.70623

**Published:** 2024-12-10

**Authors:** Bernard W. T. Coetzee, Layla van Zyl

**Affiliations:** ^1^ Conservation Ecology Research Unit, Department of Zoology and Entomology University of Pretoria Pretoria South Africa

**Keywords:** ecology, plant traits, radiance, restoration ecology

## Abstract

Light is a fundamental attribute and key abiotic driver in forest ecosystems. Although the ecological effects of light itself is well studied, capturing the complex parameters that constitute the whole light environment remain an intricate research endeavor. Here, we apply the newly introduced environmental light field (ELF) technique in Kibale National Park, Uganda. We captured whole light scenes with repeat photography and processed it to measure both the spectral composition of light in the red‐green‐blue range, as well as its variation, or “contrast‐span”, using the newly introduced International System of Units (SI); “lit”. We compare across major and globally common utilized forest types—primary, secondary, and selectively logged areas, as well as a completely cleared area as a control. We find that the ELF system is able to effectively capture key aspects of the local light environment across the range of forest types. The distribution of light intensity and its spectral composition across our study is hardly uniform, with primary forest and a clearing showing two orders of magnitude difference in light. Blue light predominates the sky areas of the clearing, indicating the Rayleigh scattering of sunlight in the atmosphere. In general, radiance decrease with increasing intactness of the forest, and selectively logged and primary forest show the most similar environmental light characteristics. Owing to its ability to capture fine scale variations in light across elevation gradients, their spectral characteristics, as well as their intensities, the ELF system should become a useful tool in better quantifying light in ecology. In particular, we discuss its potential use in restoration ecology.

## Graphical abstract

The environmental light field system excels at measuring fundamental attributes of the whole scene natural light regime in different forest types.

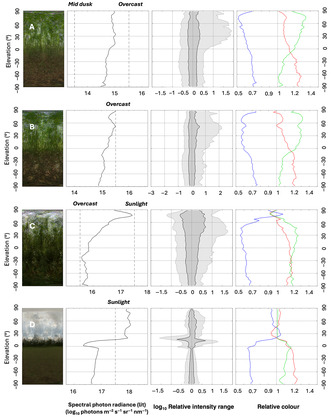

## 1 Introduction

Light is a fundamental ecological parameter. In forest systems in particular, there is a long history in identifying the factors that influence light variability, and especially how it relates to aspects of vegetation structural complexity via forest structure and canopy architecture (see Endler 1993; Coverdale and Davies 2023). This is because light plays a key role in ecological processes such as tree seedling generation (Montgomery and Chazdon 2001), regrowth of understory (Balandier et al. 2022), and it can alter physical parameters like microclimate (Valladares et al. 2016). It plays a role in phenological events such as flowering and leaf shedding (Fitter and Hay 2012) and various other the diel timing of events, such as navigation, foraging, and mating, which are crucial for maintaining population dynamics and species interactions (Endler 1993; Kronfeld‐Schor et al. 2017).

Research into the light environment has mainly focused on the mechanisms altering light, and how these may be correlated to changes in light (like vegetation structure; Coverdale and Davies 2023), rather than the characteristics of light, and the light environment, per se. We briefly overview the likely reasons for this. First, it may be because the complexity of the light environment is broadly underappreciated. Light is composed of photons, an elementary particle, and the deeply enigmatic nature of light is still not resolved in many branches of physics, let alone in ecology (Roychoudhuri, Kracklauer, and Creath 2017). Light is not simply a one dimensional phenomena rather it is composed of and can be disaggregated in, for example, components of radiance, irradiance, luminance, illuminance, spectral composition, intensity, polarization, and contrast (see Table 1 for definitions; Land and Nilsson 2012). The spatial patterning of the light environment is not simply a consequence of canopy and sub‐canopy vegetation orientation, but rather a complex interaction between various structures, their textures and colors (Montgomery and Chazdon 2001). An obvious example of this oversight is the widespread use of luxmeters to measure light intensity. Lux meters measure illuminance with an upward facing cosine corrected sensor, thus mainly quantifying light that reaches the environment from above. However, light in the environment has been reflected and refracted or been absorbed. This is fundamentally important—ecologically relevant light does not only consist of a light source but rather the materials, objects, and surfaces that are being illuminated (Nilsson and Smolka 2021).

**TABLE 1:** Glossary of terms pertaining to the different components of light that constitute the whole light environment.

Term	Definition
Radiance	The radiant flux emitted, reflected, transmitted, or received by a surface per unit solid angle per unit projected area. It quantifies the brightness of a surface in a given direction.
Irradiance	The radiant flux incident on a surface per unit area. It is measured in watts per square meter (W/m^2^). Irradiance indicates the amount of radiant energy received by a surface.
Luminance	The measure of the intensity of light emitted or reflected from a surface in a particular direction. It is often measured in candelas per square meter (cd/m^2^). Luminance is used to describe the brightness of a surface as perceived by the human eye.
Illuminance	The measure of the amount of light incident on a surface. It is measured in lux (lx). Illuminance describes the brightness of an illuminated surface as perceived by an observer.
Spectral composition	The distribution of light energy across different wavelengths in the electromagnetic spectrum. It describes the relative intensities of different wavelengths present in a light source.
Intensity	Intensity refers to the amount of light energy emitted, transmitted, or received in a particular direction. It is often used interchangeably with radiance or irradiance, depending on the context.
Polarization	The orientation of the oscillations of light waves in a particular direction. Polarized light waves vibrate in a specific plane perpendicular to the direction of propagation. Polarization can be linear, circular, or elliptical.
Contrast	Contrast is the difference in luminance or color that makes an object distinguishable from its background. In images or visual displays, contrast refers to the distinction between light and dark areas or between different colors. High contrast means there is a significant difference between adjacent elements, while low contrast means there is little difference.

Second, the study of the light environment has been hampered by the lack of suitable tools or at least by the use of tools that have been primarily designed for other purposes than ecological applications. Spherical densiometers, first introduced in 1956 (Lemmon 1956), are still in use, despite revealing little about the characteristics of the light other than an index of tree cover. When correlated with five forest monitoring smartphone apps, many of which use phone cameras to estimate canopy cover, the canopy openness and ground cover show weak correlations to densiometers readings (Schweizer et al. 2024), although the direction of the causality is unclear. Photon flux meters are widely used and useful (Montgomery and Chazdon 2001; Matsuo et al. 2021), but still only provide a small snapshot of the characteristics of the light, as they measure photons over a relatively small area. Spectrometers can provide spectral information of light, but still only limited inference on the nature of the light, are expensive, and hard to use at scale (but see developments in miniaturization—i.e., Troscianko 2023). Simple hemispheric photography can replicate a densiometer and ease data collection and processing (Helbach et al. 2022), but captures no information on the characteristics of the light itself.

Furthering our understanding into the relationships between the light environment, and ecologically and economically important functioning of ecosystems, may be of particular importance to restoration ecology. Restoration ecology is in part concerned with returning degraded ecosystem structure, function, and composition to a target baseline, but which parameters the most important, how to achieve them, and how to measure them, still remains a challenge to the field (Perring et al. 2015). Conceptually, if the light characteristics of a particular degraded patch can be quantified, and shown similar to that of a natural baseline area, it can be argued that at least some structural characteristics of that patch is restored. However, a description of the light characteristics itself is needed before any predictions on the restoration value of replicating vegetation structure can be made. Although multiple techniques now excel at capturing structural information, especially with advanced laser altimetry, commonly called light detection and ranging (LIDAR; Vierling et al. 2008), the detection of the light environment itself, and how those characteristics may change ecological patterns and processes remains to be resolved.

**FIGURE 1:**
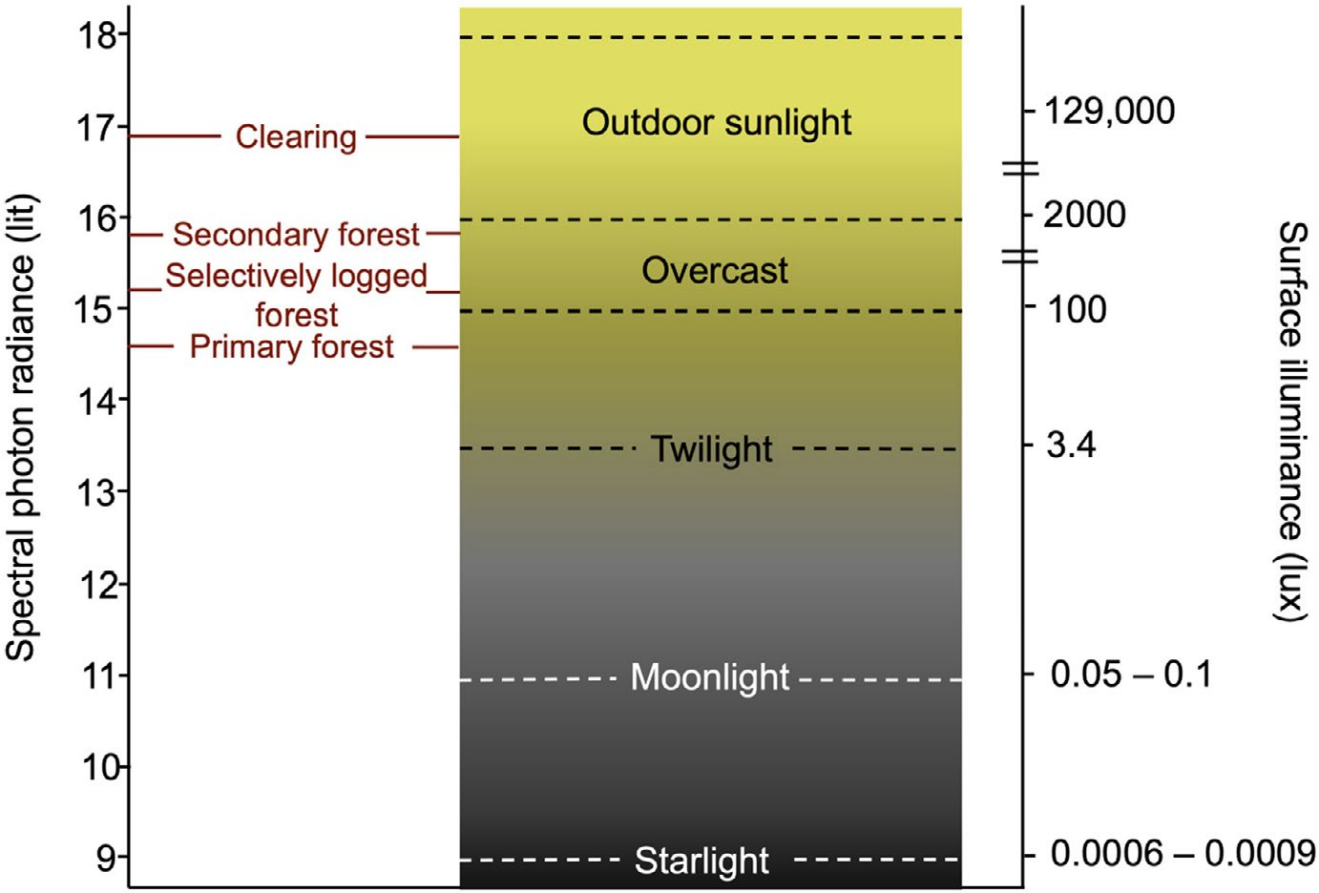
Conceptual diagram showing the spectral photon radiance (i.e., absolute intensity) (lit) as well as surface illuminance (lux) of different natural lighting environments. Median white‐light spectral photon radiance (lit) of each forest type (Primary Forest, selectively logged forest, secondary forest, clearing) indicated for comparison with known natural lighting environments (surface illuminance values from Hänel et al. 2018). Note that “lit” and “lux” are provided for reference for readers that may be more familiar with “lux”, but that the two units cannot be directly compared, since “lit” measures a whole scene, while “lux” is essentially only a point source measurement.

One potential solution to measuring ecologically relevant light is whole scene photography. The environmental light field (ELF) technique was introduced in 2021, and is set to revolutionize how ecologists measure the characteristics of the whole light environment (Nilsson and Smolka 2021). It is essentially a photography system, using a spherical lens, which more fully captures the different parameters that constitute the whole light environment. The ELF method uses repeat photography, with a calibrated digital single lens reflex camera and a wide‐angle spherical lens combination, to record radiances as a function of elevation angle. Significantly, the technique has introduced a new SI unit, the absolute photon flux (“lit”; Figure 1), that quantifies the spectral composition in the red‐green‐blue range, as well as its variation or “contrast‐span”. Here, we apply the ELF technique across a range of forest types (primary forest, secondary forest, selectively logged forest, and clearing) in Kibale National Park, Uganda. In doing so, we aim to measure whole scene environment characteristics of divergent forest structures, to test if this technique could be applied more broadly into the study of ecological light. We discuss also how this may be achieved. We hypothesize that the ELF system can discriminate key features of the whole scene light environment, with clear differences in the forest types.

## 2 Methods

### 2.1 Study Site and Forest Types

The study was carried out in three forest types in Kibale National Park, Uganda, close to the Makerere University Biological Field station (N0.562, E30.356). Kibale National Park spans approximately 766 km^2^, contains both lowland and evergreen tropical montane forests with elevation ranges from 1100 to 1600 m. The region experiences two rainy seasons, from March to May and September to November, and two dry seasons, from December to February and June to August. The average annual rainfall in Kibale National Park is 1749 mm. The park is renowned for hosting 13 species of primates, along with other large mammals such as elephants (Chapman, Struhsaker, and Lambert 2005).

Light measurements (detail below) where conducted in four different forest types, namely primary forest, selectively logged forest, secondary forest, and a clearing. The primary forest area has never been logged or otherwise disturbed, is minimally impacted by other forms of disturbance, and while occasional human visits occur in the form of researchers, illegal poachers and anti‐poaching activities, is otherwise rarely visited by people. It is characterized by an intact canopy and relative open understory (Figure S1; N0.56058, E30.36155). Selective logging took place in Kibale National Park from 1968 to 1969 (Figure S2; Latja et al. 2016; N0.57032,). Selective logging removes selected trees based on criteria such as diameter, height, or species. Remaining trees are left in the stand, as opposed to clearcutting where all trees are felled. The secondary forest was intensely logged historically from about 1987, but since 2004 has not been logged (Figure S3; N0.56802, E30.35858). The secondary forest has regenerated after this disturbance. It is characterized by younger trees, a mature understory, and complex tree structures, and altered biodiversity. The clearing is a football pitch (approximately 60 m × 100 m in total; Figure S4; N0.5564, E30.3547) and essentially acts as a baseline or control in our study, typifying the light environment in the region in the absence of any vegetation.

### 2.2 Light Measurements

The full methods for the environmental light field (ELF) technique and analysis are described in Nilsson and Smolka (2021). In brief, we captured the light characteristics from 14 to 17 August 2023 across the four forest types described above (“environments” in ELF vernacular). In each environment, we captured one image of four “scenes” (in ELF vernacular). Each environment is quantified by four sequences of photographs. Each photograph is bracketed by three full exposure evaluation settings (3EV), to better capture the full range of both underexposed and overexposed light (a “scene”). Bracketing involves three exposures for each image, each separated by three EV units (thus spanning six EV units). In consequence, we processed a total of 48 photos (from 4 forest types [environments], four scenes each, and 3EV per scenes [4 × 4 × 3]). This represents an “exhaustive” approach—depending on the research question, just one set of environmental photographs is sufficient to describe many of the parameters of the light environment, and so our approach better captures a suite of potential variation. We note that using several random points in each forest type would produce smoother curves by averaging out scene‐specific variation that are of no relevance.

At each forest type, we used a Nikon D850 and a Sigma 8 mm 180° spherical lens, both of which are calibrated and used in conjunction with equipment specific calibration data (Nilsson and Smolka 2021; see Figure S5). The camera is set up 1 m above the ground on a sturdy tripod, and leveled on all directions of the horizontal plane using a bubble level. After a scene is taken, the camera is rotated 90° in a clockwise direction, to measure overlap between scenes, the camera is leveled again. Four such scenes, bracketed by 3EV at each, constitutes one measurement of the light environment. For comparative purposes, and to reduce differences in light due to the position of the sun, we aimed to complete all scenes as close to noon as logistically feasible (median = 10 min; *N* = 48), and under identical weather conditions (all partly cloudy). Because these measurements are so data rich, and characterize the light environment in its entirety, replication within environments are not required. Indeed, within forest types, there was no significant difference when repeating this methodology in different but similarly logged areas, either in overall spectral composition or contrast span (data not shown). Our approach is akin, for example, to the use of ground‐based LIDAR (Vierling et al. 2008).

### 2.3 Data Analysis

Data was processed with the Environmental Light Field Software Toolbox in Matlab (Nilsson and Smolka 2021). For each environment, each set of scenes are stretched from the hemispherical circular image of the spherical lens to a re‐mapped square grid on a 0°–180° azimuth *x*‐axis and a −90° to +90° elevation *y*‐axis. While this may distort the solid angles in the upper and narrow extremes of the image, it has no consequence on the mathematical weighting across elevations. Thereafter, all images are stacked, and radiance data processed displayed as an average. Each image contains radiance data on the light distribution of a scene, which produces representative measurements for arbitrary positions in the environment, and allows specific features of individual scenes to be removed by averaging (e.g., overexposed sections from a bright light source like the sun). The human and animal eye has a spectral resolution similar to the typical sample bandwidths of RGB image sensor (50–120 nm half‐widths). In consequence, the radiances are calculated separately in the red, green, and blue spectral channels, as well as the integrated channel (i.e., white light). The data is expressed in the newly introduced SI unit of “photon flux radiance” (“lit”), which is based on the photon flux per area, solid angle, and the spectral range (photons s^−1^ m^−2^ sr^−1^ nm^−1^). Given the nine orders of magnitude difference from starlight to sunlight, the log_10_ values are used to describe radiance (or the “lit” unit; the log_10_ number of photons per second per square meter per steradian per nanometer wavelength). For practical interpretation, Figure 1 compares the radiance values across different natural light regimes. We present both a simplified table, and more data‐rich visual representation, of all radiance values across forest types.

## 3 Results

**FIGURE 2:**
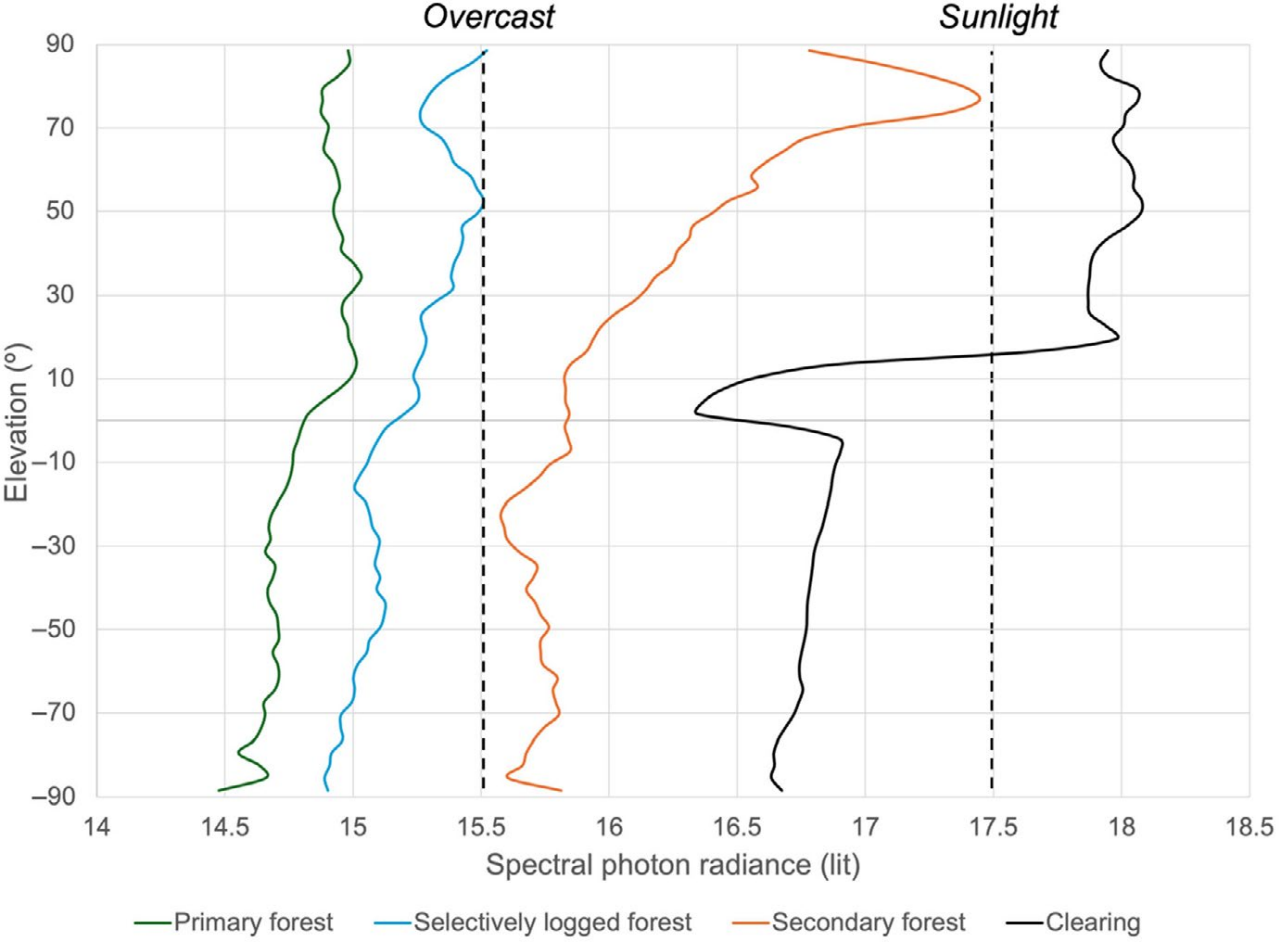
Median spectral photon radiance of white‐light (i.e., absolute intensity) (lit) as a function of elevation (°) in the various forest types (Primary forest, selectively logged forest, secondary forest and clearing).

In general, the environmental light field (ELF) system is able to characterize and capture key aspects of the local light environment across the range of sites in our study system. As could be expected, the distribution of light intensity and its spectral composition across our study is hardly uniform. At the extremes, the forest clearing displays a median lit value of 16.9 ± 9.2, which can be characterized as “Sunlight”, whereas the primary forest has a median lit value of 14.8 ± 8, which can be characterized as “Overcast” (Table 2; Figure 2). It should be noted that because the lit unit is on a logarithmic scale, this represents two orders of magnitude change in the overall radiance at the extremes. Selectively logged forest is only slightly darker than secondary forest (~0.7 lit). The ELF system also captures the elevation changes in light accurately to expectation—the higher, and more open regions of the canopy indeed display higher radiance values. In general, radiance decrease with increasing intactness of the forest, and selectively logged and primary forest show the most similar environmental light characteristics.

**TABLE 2:** Simplified representation of ELF data, showing the median spectral photon radiance (lit) of white‐light in the respective forest types (bold), with their associated contrast‐span with 95% of all values. Spectral composition is shown by the relative contributions (%) of the red (R), green (G) and blue (B) spectral channels.

Forest type			
Primary	**14.8** ± 8.0 lit		
	R 39.0%	G 39.8%	B 21.2%
Selectively logged forest	**15.2** ± 8.4 lit		
	R 38.6%	G 40.6%	B 20.8%
Secondary forest	**15.9** ± 8.9 lit		
	R 38.4%	G 41.1%	B 20.5%
Clearing	**16.9** ± 9.2 lit		
	R 40.1%	G 37.4%	B 22.5%

**FIGURE 3:**
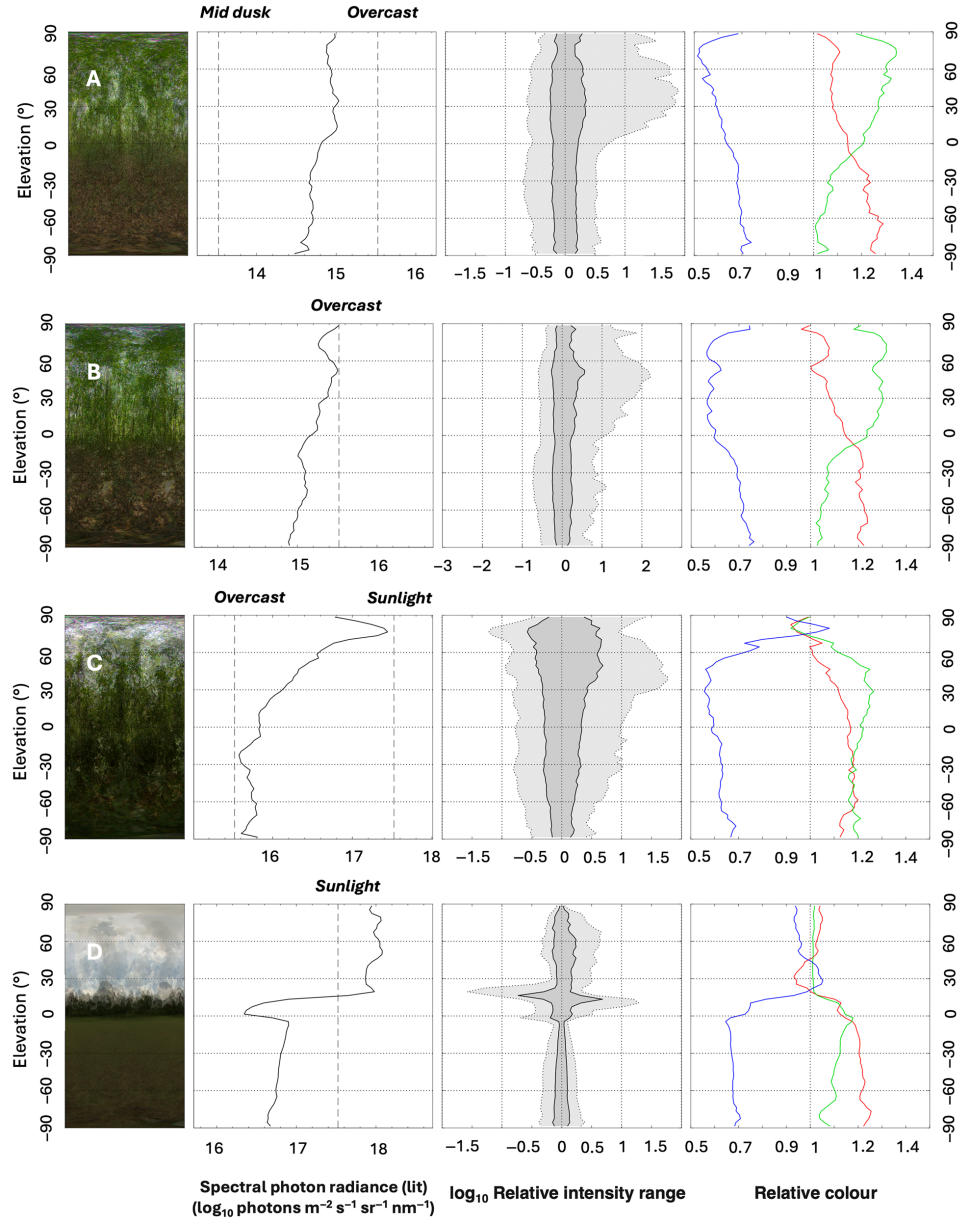
Whole scene light environments as described by the environmental light field technique in (A) primary forest, (B) selectively logged forest, (C) secondary forest and (D) clearing, in and around Kibale National Park, Uganda. Apart from the large differences in absolute radiance, the curves also reveal systematic differences in spectral composition and relative radiance distribution at different elevation angles, and the unique light characteristics of each forest type. Note the order of magnitude changes in the *x*‐axis. The dashed levels of overcast and sunlight are provided to aid interpreting on the radiance scale. See also Figure 1 for aid in interpretation.

Furthermore, the clearing shows a large change in overall radiance at 0° elevation, or as one measures from sky towards the ground. The forested regions show more similar profiles in spectral photon radiance such as higher radiances at the canopy than the ground, but with some important deviations. Because the canopy of the logged forests is more open, light in the upper elevations, show a 1.5× decline in spectral photon radiance from the upper elevations to around 80°, as the canopy starts to close (Figure 3). Such a change is not as evident in selectively logged and primary forest, ostensibly because the canopy in those regimes is more intact and similar in terms of structure.

The relative intensity range is the range of radiances or the contrast‐span for white light, for 50% (dark gray) and 95% (light gray) confidence intervals, across elevation gradients in processed images (Figure 3). In more simple terms, it is the range of brightness in an image. For reference, in the clearing the sky is uniformly light, and the grass below elevation of 0° is uniformly dark, and so in either elevation the intensity range is narrower. However, in the region where its imaging distant forest from side one, so from ≈0°–25°, there is a range of radiances as light is differentially filtered, absorbed and scattered and so the contrast span in that region is higher. The contrast intensity ranges in forests are broadly similar, despite having different relative spectral characteristics, although the logged forest shows variation in relative intensity range (Figure 3).

The ELF system is adept at capturing the spectral differences between forest types. Blue light predominates the sky areas of the clearing, indicating the Rayleigh scattering of sunlight in the atmosphere. All forest types share a similar blue light color profile, even across elevational bands. In the clearing, there is little green color detected above ≈25°, where no green vegetation and only clear sky is photographed. The understory of the selectively logged and primary forest in our study region is fairly open and characterized by mainly dry and drying leaf matter, with some bare soil patches, and indeed the relative color diagram reflects this lack of green material. Conversely, the logged forest has a rich and dense understory of smaller shrubs and trees, which is well reflected across the green channel from an elevation angle of +80° downwards.

## 4 Discussion

The environmental light field (ELF) system excels at measuring fundamental attributes of the whole scene natural light regime in different forest types. It captures a range of spectral characteristics, intensities and contrast spans, and the differences reflect alterations to the canopy structure of the different forest types. Open areas show higher intensities of light predominantly in the blue channel, and where the forest understories are more mature, the green channel is higher.

These results inform a variety of aspects into the ecology of the forest in Kibale National Park. As could be expected, the light regimes in selectively logged forest more closely mimic that of primary forest, while secondary forest is more lit. Because forest structure strongly mediates vegetation response to light, and so can impact functional traits and total plant cover (De Pauw et al. 2022), these results can aid understanding into how such changes in light regimes may influence forest regeneration. The lower levels of green channel in primary and selectively logged forest at lower elevations indicate less green vegetation structure in the understory, conforming with expectations that overstory cover can significantly affect understory cover (Krebs, Reeves, and Baggett 2019). The higher contrast span in primary forest at higher elevations captures the absorption of light in this mature canopy, emphasizing that the local light environment results from a complex interaction among various structures, textures, and colors, rather than merely the orientation of canopy and sub‐canopy vegetation (Montgomery and Chazdon 2001).

The role of light in forest restoration ecology is well appreciated (Endler 1993; Messier et al. 1999; Funk and McDaniel 2010), although rather from the perspective of its impact on regeneration, rather what constitutes the light characteristics of a base‐line ecosystem. Light can play a critical role in better understanding restoration ecology, as it influences the success and sustainability of ecosystem recovery efforts. Light availability affects species composition and diversity, as different plants have varying traits that influence their light requirements and tolerances (Valladares et al. 2016). Understanding and managing light conditions can help restore natural plant communities, promoting the establishment of native species while suppressing invasive ones. For example, to establish the endangered endemic Hawaiian fern *Marsilea villosa* , the interaction of weed and flood management is critical, but also ensuring conditions of moderate shade (Chau and Reyes 2014). Microsite variations in light were positively correlated with growth, but not survival in the upland understory regeneration in Thailand (Sangsupan et al. 2021). However, the outcomes of light can be highly context specific—dispersal, not understory light competition, limits restoration of Iowa Woodland understory (Brudvig, Mabry, and Mottl 2011). Nonetheless, a better understanding of the whole habitats environmental light characteristics will aid in comparing different restoration stages directly with each other and provide at a minimum a dimension on the structural characteristics of that habitat. Owing to its ability to capture fine scale variations in light across elevation gradients, their spectral characteristics, as well as their intensities, the ELF system should become a useful tool in restoration ecology.

More generally, the variation in the vertical gradients of radiance and spatial structure (contrast) are likely to have a strong impact on animal behavior and spatial distribution (reviewed by Nilsson, Smolka, and Bok 2022). Species‐specific responses to vertical light gradients are likely to play a crucial role in defining the ecological niches of various species (Ausprey, Newell, and Robinson 2021), how this may alter species traits (Ausprey 2021), as well as fine scale variation in niche partitioning (Gerrish et al. 2009). Investigating these responses will introduce a new dimension to behavioral ecology and enhance our understanding of the mechanisms that drive habitat selection and activity patterns across a suite of species. Furthermore, such variations in light driven by habitat structure are critical for understating and predicting plant growth. For example, blue light is well characterized by the ELF system, with the more open systems being exposed to more than more closed systems. Blue light is a well‐known stimulant of phytotropins, a key factor for predicting phototropism, chloroplast movement, leaf expansion, and stomatal opening (Takemiya et al. 2005). In consequence, the ELF system could potentially be operationalized to predict the relationships between habitat structure and understory growth rates.

Measuring light in forests has important implications for forest management, as light directly influences tree growth, forest structure, biodiversity, and overall ecosystem health. Understanding these dynamics could help forest managers make decisions about tree thinning or planting that can promote desired species. Light measurement also plays a crucial role in assessing forest productivity and carbon sequestration potential, as more light supports higher photosynthesis and faster tree growth, contributing to carbon absorption for climate mitigation. With climate change impacting forest ecosystems, regularly measuring light help could help track changes in canopy density and growth patterns, see for instance Mizunuma et al. (2013).

The ELF system represents an advancement in whole scene representation of light, but challenges remain in measuring light for the ecologist. It should be noted that in general, the spectral sensitivity of a camera's RGB channels are not ideal for acquiring precise radiometric data, as the spectral curves of the individual channels are bell shaped (with different sensitivity to different wavelengths) and there can be overlap between the channels. However, the error in individual RGB channels may exceed 0.1–0.2 lit, and so non‐ideal spectral sensitivity of typical RGB image‐sensors are thus small compared to the radiance range within scenes and the variation between different environments (Nilsson and Smolka 2021). However, if the goal of study is to measure the precise spectral composition of light that reaches a leaf for instance, a more suitable tool for purpose should be used, such as a spectroradiometer.

In addition, the implementation of the ELF system used here is optimized for the human photopic system, and so does not measure ultraviolet light, which is of particular importance to especially birds and insects—although at least conceptually, UV pass cameras could be used to resolve this (Nilsson and Smolka 2021) and simple procedures can add UV to ELF measurements (see Vasdal et al. 2022). A further requirement is for sensitivity to extremely low light levels across the ultraviolet to human‐visible spectrum. Minatare spectroradiometers provide a cost‐effective method for measuring spectral radiance and irradiance in the UV‐A to near‐infrared range, even at low light levels. This capability makes them ideal for behavioral and ecological studies, including research on artificial light at night (ALAN; Troscianko 2023). In a recent study, which examined the impact of lighting on the “landscape of fear” in an endangered shorebird, the use of such devices demonstrated that ALAN can shape the landscape of fear and interacts with optimal foraging decisions (Jolkkonen, Gaston, and Troscianko 2023). In consequences, key advances in understanding the ecology of light, and drivers of change such as ALAN, can be made by combining such technologies, understanding their strengths and weaknesses, and complementarity.

We did not directly measure the structure of the vegetation across our study sites. While these results cannot directly measure forest structure, the light regimes do indicate the expected relationship with it, where denser, more mature canopies allow less light to penetrate to ground level. Future work could be usefully directed at building a repository of how classical measurements of vegetation structure correlate with the whole light scene environments (Acar and Osman 2022). By replication, and by repeating both the light and vegetation structure measurements across different habitat and vegetation types, and habitat modification regimes, a repository could usefully be built over time, and regression analysis be used to predict light regimes in various habitat types.

In conclusion, the environmental light field (ELF) system effectively measures natural light regimes, revealing how canopy structures influence light distribution in forests. This system could prove invaluable for restoration ecology, as light availability affects species composition and ecosystem recovery. While the ELF system has limitations in fine scale spectral measurement and does not yet capture UV light, it still provides significant insights into habitat light characteristics. Taken together, it means the ELF system could usefully be applied to advance our understanding of environmental light in ecology, with practical applications for conservation.

## Author Contributions

**Bernard W. T. Coetzee:** conceptualization (equal), data curation (equal), formal analysis (equal), funding acquisition (equal), investigation (equal), methodology (equal), project administration (equal), supervision (equal), validation (equal), visualization (equal), writing – original draft (equal), writing – review and editing (equal). **Layla van Zyl:** data curation (equal), formal analysis (equal), methodology (equal), visualization (equal), writing – review and editing (equal).

## Conflicts of Interest

The authors declare no conflicts of interest.

## Supporting information


Appendix S1

## Data Availability

All raw data are here: https://doi.org/10.5061/dryad.3xsj3txr6. Reviewer URL: http://datadryad.org/stash/share/2fsOUNRQG0GzeIXOq8dsClnG6Boc1CYdewxtB0JeSkc.
