# Supplementary material for: The Environmental Light Characteristics of Forest Under Different Logging Regimes
Source: Ecol Evol. 2024 Dec 10;14(12):e70623. doi: 10.1002/ece3.70623 (PMC11631710; doi:10.1002/ece3.70623)
Supplement: Supplementary file 1 — Appendix S1 [file ECE3-14-e70623-s001.docx]

**Supplementary material**


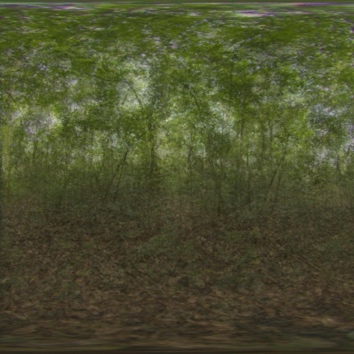
 **A**


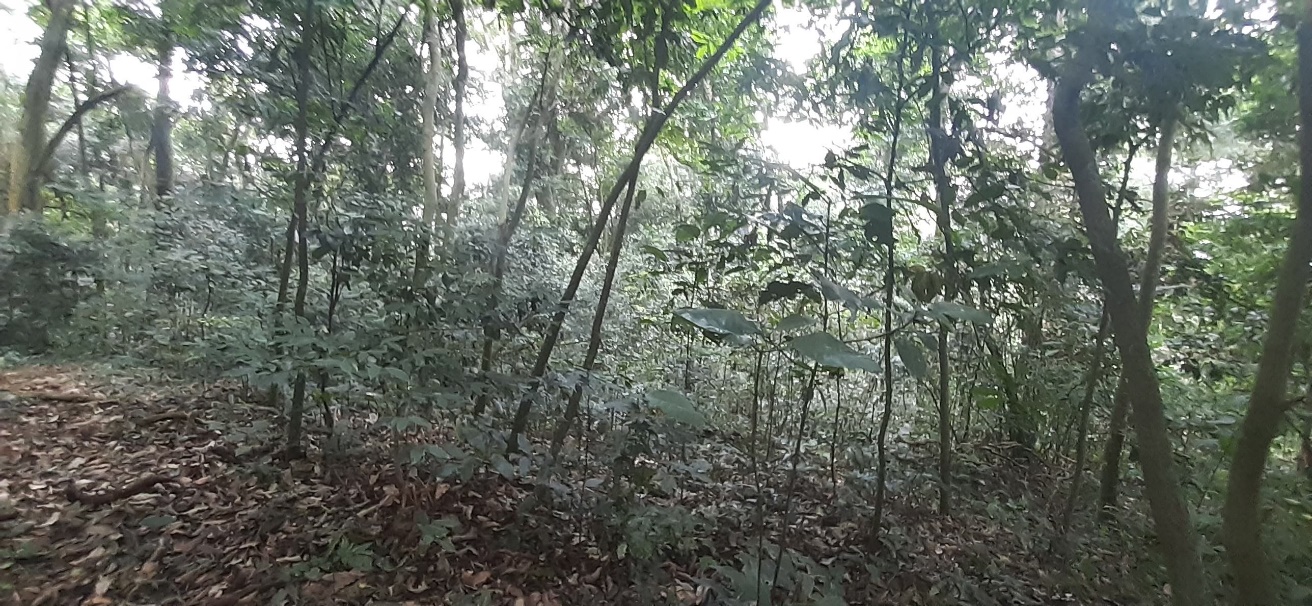
 **B**

**Figure S1.** Composite image (A) and site picture (B) of the primary forest in Kibale National Park, Uganda.


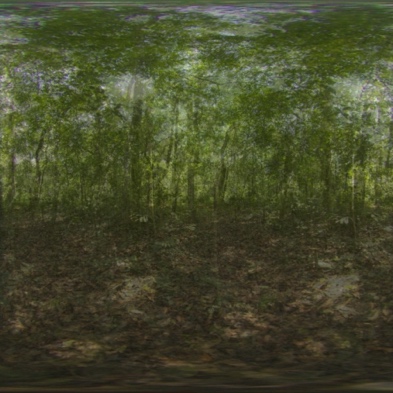
**A**


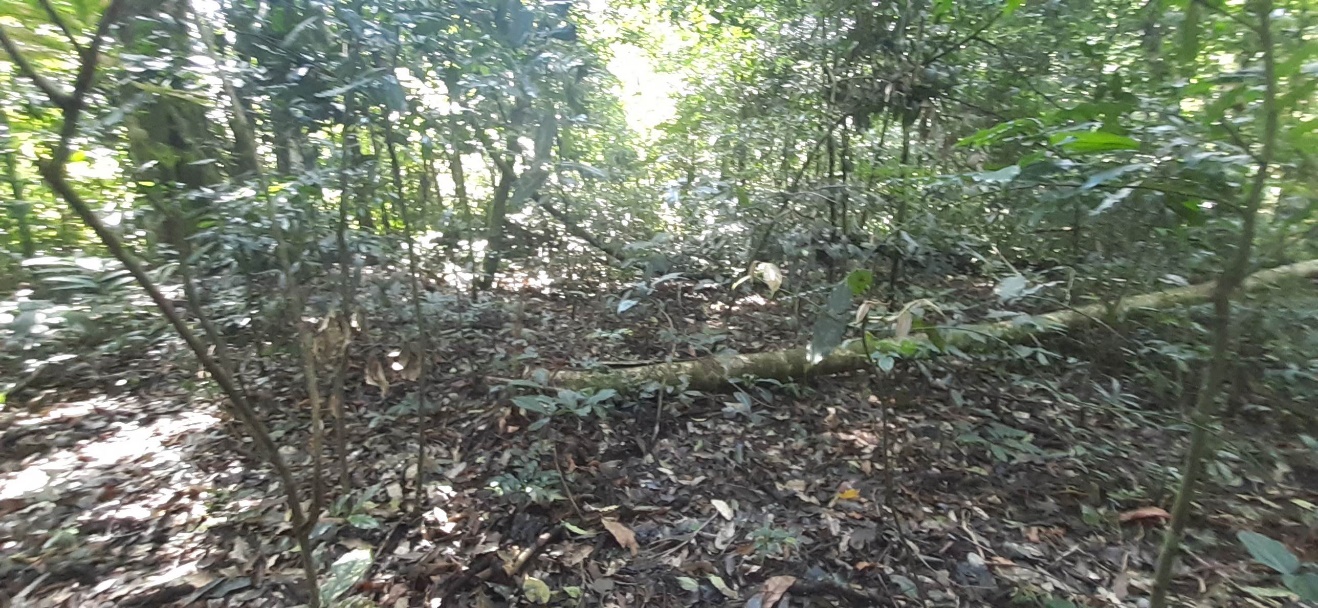
 **B**

**Figure S2.** Composite image (A) and site picture (B) of the selectively logged forest in Kibale National Park, Uganda.


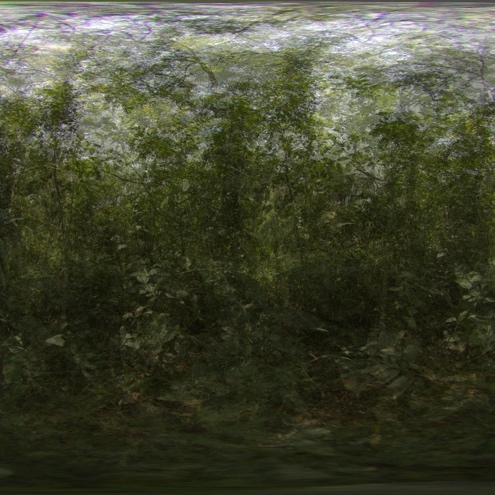
**A**


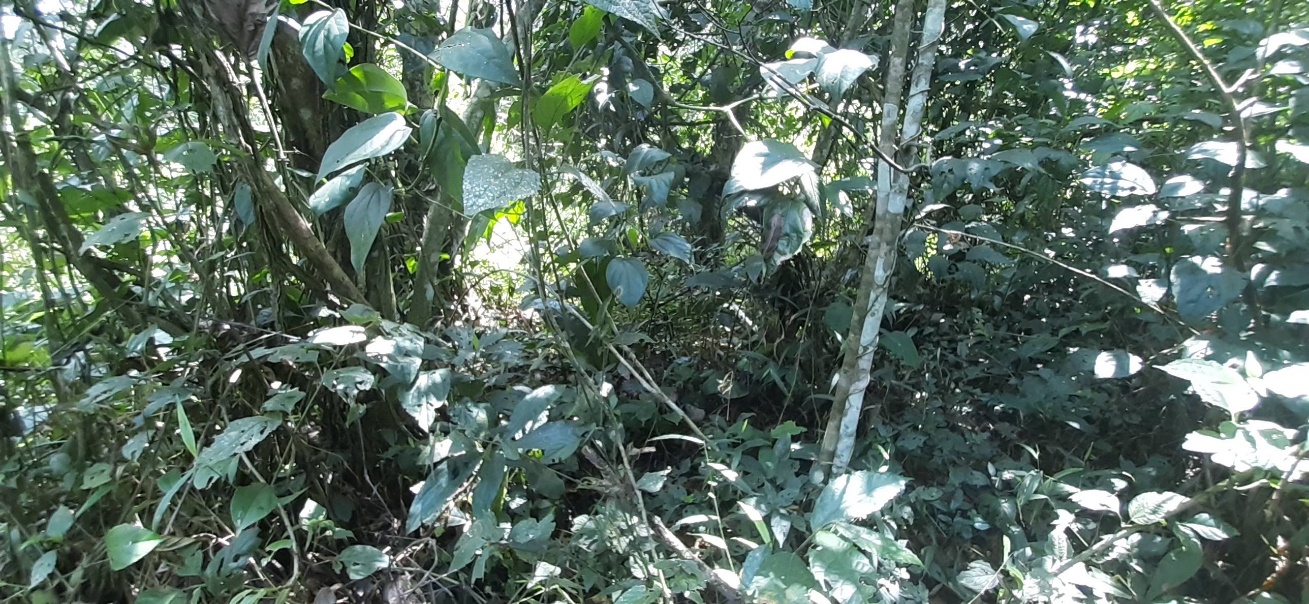
 **B**

**Figure S3.** Composite image (A) and site picture (B) of the secondary logged forest in Kibale National Park, Uganda.


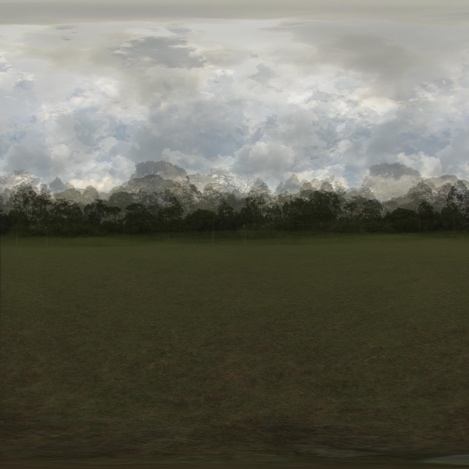
**A**


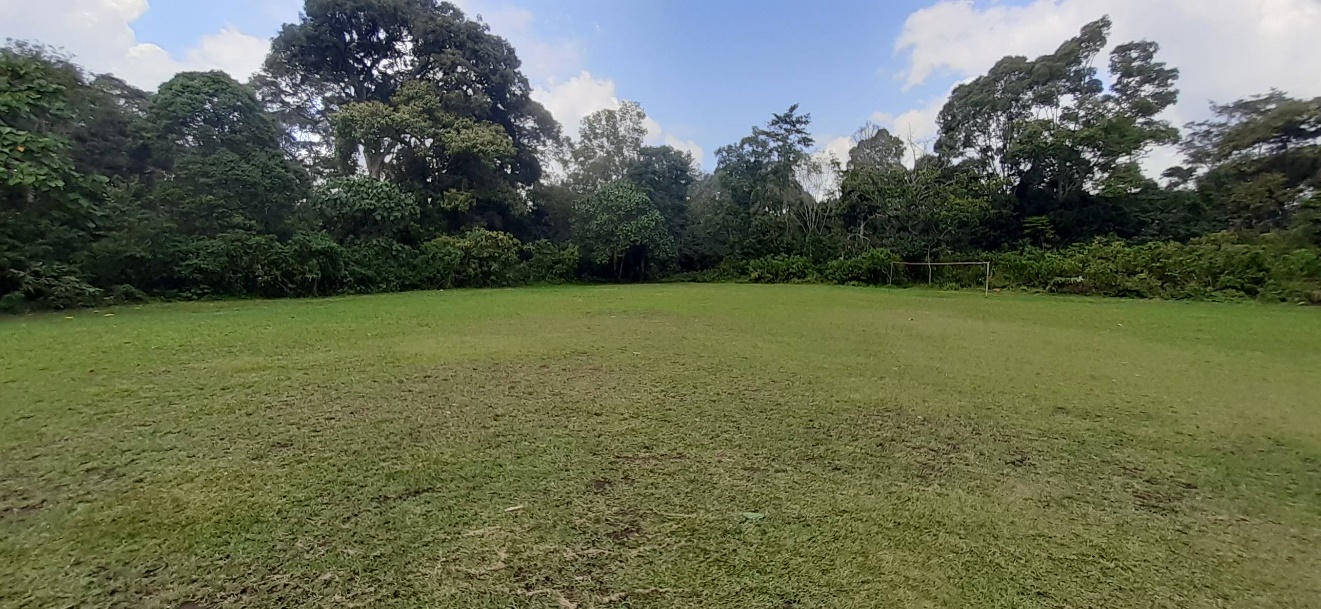
**B**

**Figure S4.** Composite image (A) and site picture (B) of a clearing outside of Kibale National Park, Uganda.


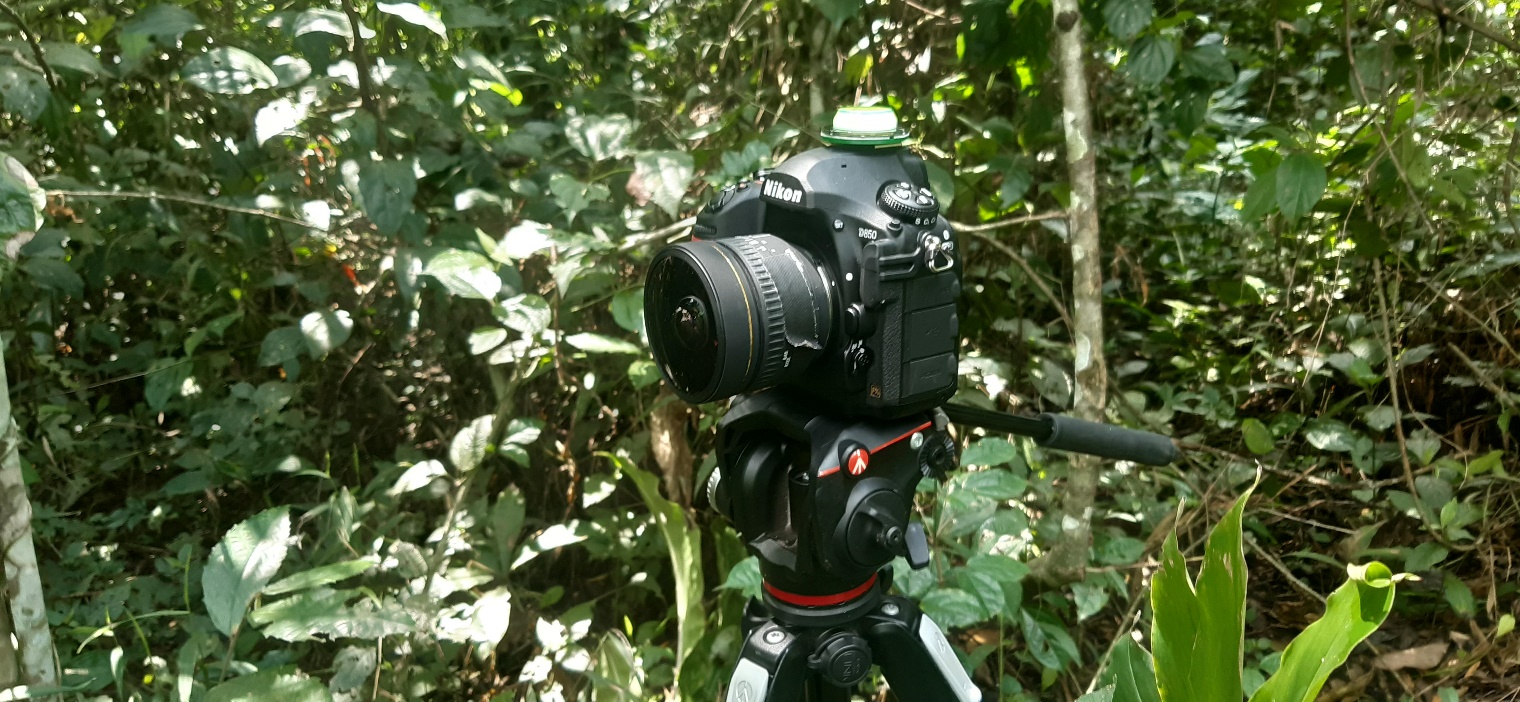


**Figure S5.** The Environmental Light Field camera deployed in the field. Note the sturdy tripod on a head that can swivel, masking tape to keep the focus ring locked and identical at all sites, the round spirit level, and the spherical 8mm spherical lens.

**--- End ---**
